# Object and action naming in adults and aged people with Down syndrome

**DOI:** 10.1590/1980-5764-DN-2024-0272

**Published:** 2025-08-11

**Authors:** Cláudia Lopes Carvalho, Aline de Souza Gonçalves Gomes da Conceição, Andressa Rodrigues Ramos, Livea Carla Fidalgo Garcez Sant’Ana, Maria de Fátima Rebouças da Silva, Octávio Gonçalves Ribeiro, Orestes Vicente Forlenza, Marcia Radanovic

**Affiliations:** 1 Universidade de São Paulo, Faculdade de Medicina, Hospital das Clínicas, Instituto de Psiquiatria, Laboratório de Neurociências, São Paulo SP, Brazil.

**Keywords:** Down Syndrome, Language Tests, Speech Therapy, Intellectual Disability, Síndrome de Down, Testes de Linguagem, Fonoterapia, Deficiência Intelectual

## Abstract

Down syndrome (DS) is a genetic condition associated with cognitive and linguistic impairments, presenting specific challenges in aging and an increased risk of cognitive decline.

**Objective:**

To evaluate naming alterations in adults and older individuals with DS using the Object and Action Naming Battery (OANB) and to identify the influence of age, educational attainment, and degree of intellectual disability (ID) on linguistic performance.

**Methods:**

This study included 26 individuals diagnosed with DS aged ≥20 years, assessed with the Cambridge Examination for Mental Disorders of Older Persons with Down Syndrome and Other People with Intellectual Disabilities (CAMDEX-DS) for cognitive evaluation and the OANB, a visual confrontation naming test including 162 objects and one hundred actions.

**Results:**

No significant differences were found between groups based on ID level in object and action naming. Literate individuals performed better than non-literates in object naming (p=0.033), while verb naming was not influenced by educational level. Age did not correlate with naming performance, suggesting that typical aging is not an isolated determinant of naming impairment.

**Conclusion:**

Our results indicate that contextual factors, such as educational level, have a greater impact on linguistic performance than intrinsic factors, such as ID degree or age. The OANB proved to be an effective tool for identifying performance patterns and supporting interventions for DS persons.

## RESUMO

A síndrome de Down (SD) é uma condição genética associada a alterações cognitivas e linguísticas cuja população apresenta desafios específicos nos cuidados com relação ao envelhecimento e maior risco de declínio cognitivo.

**Objetivo:**

Avaliar alterações na nomeação em adultos e idosos com SD utilizando a bateria de nomeação de objetos e verbos (BNOV) e identificar a influência de idade, escolaridade e grau de deficiência intelectual (DI) no desempenho linguístico.

**Métodos:**

Participaram do estudo 26 indivíduos com diagnóstico de SD e idade ≥20 anos, avaliados pelo Cambridge Examination for Mental Disorders of Older Persons with Down Syndrome and Other People with Intellectual Disabilities para aspectos cognitivos e pela BNOV, um teste de nomeação por confrontação visual composto de 162 objetos e cem ações.

**Resultados:**

Não houve diferenças significativas na nomeação de substantivos e verbos entre os grupos de acordo com o grau de DI. Indivíduos alfabetizados apresentaram desempenho superior na nomeação de objetos (p=0,033) em relação aos não alfabetizados, enquanto a nomeação de verbos não foi influenciada pela escolaridade. A idade não se correlacionou com o desempenho na nomeação, o que sugere que o envelhecimento isoladamente não seja determinante de declínio nessa tarefa.

**Conclusão:**

Fatores contextuais, como escolaridade, mostraram produzir maior impacto no desempenho linguístico do que fatores intrínsecos, como grau de DI ou idade. A BNOV demonstrou ser uma ferramenta útil para identificar padrões de desempenho e subsidiar intervenções para populações com SD.

## INTRODUCTION

Down syndrome (DS) is a genetic condition characterized by the presence of an extra copy of chromosome 21, resulting in a series of clinical, cognitive, and behavioral alterations, with intellectual disability (ID) being the most striking feature of this condition. DS is estimated to be the second leading cause of ID among live births, with a significant impact on the development of cognitive and language skills. Language development is often delayed, with impairment more pronounced in expression than in language comprehension^1^. The language delay translates into difficulties such as phonological disorders, simplification in sentence structure, reduced length of utterances, syntactic errors, difficulties in using auxiliary verbs, poor elaboration of conversational speech, and the presence of apraxia of speech^2^. These linguistic alterations persist throughout life, affecting the communication of adults and older people with DS^3^.

In recent years, advances in medicine and health care have led to a significant increase in the life expectancy of people with DS, reaching approximately six decades^4^. However, as this population ages, clinical manifestations associated with premature aging appear, including an increased risk of developing dementia, especially Alzheimer’s disease (AD)^5^. Trisomy 21 generates an overexpression of genes encoding the amyloid precursor protein (APP) (whose cleavage generates the beta-amyloid peptide implicated in the pathogenesis of AD)^6^. Thus, DS presents a unique scenario in studying cognitive aging due to the overlap of previous cognitive impairments associated with an increased risk of neurodegenerative diseases^7^.

Assessing language changes in adults and older adults with DS, especially in the context of aging, is a clinical challenge, particularly due to the difficulty in distinguishing normal language declines in aging from those caused by AD. Naming is a complex linguistic function that is essential for effective communication, requiring the retrieval of phonological and semantic information stored in the memory system and accessed when faced with a stimulus^8^, and is one of the processes most affected in cognitive disorders such as dementia^9^. Structured tools, such as visual confrontation naming tests, are essential to accurately assess the naming process, since anomia is a common symptom in patients with cognitive impairment.

This study aims to enhance our understanding of naming alterations in adults and aging persons with DS, using a visual confrontation naming test that assesses both object (noun) and action (verb) naming to identify and quantify these difficulties. Our data provides a perspective of this population’s particularities regarding naming abilities and contributes to improving clinical practices in the diagnosis and therapeutic management of their linguistic alterations.

## METHODS

This cross-sectional descriptive study was conducted at an outpatient specialized clinic for people with DS linked to a university hospital. The project received approval from the local and National Research Ethics Committees (CONEP: 148611/2017). All research participants and/or their legal guardians provided signed informed consent.

### Inclusion criteria

We recruited 26 individuals of both sexes who met the International Classification of Diseases 10^th^ Revision (ICD-10) diagnostic criteria for DS (Q.90). These individuals were native Brazilian Portuguese speakers aged 20 or older. Participants were required to have maintained stable cognitive and behavioral status for the past five years, as assessed by a standardized structured instrument designed to facilitate the diagnosis of dementia in people with DS and differentiate decline due to dementia or other mental disorders from pre-existing impairment (see Cognitive assessment) to be considered as “without cognitive impairment” and enrolled in the study.

### Exclusion criteria

- Subjective complaints or objective evidence of hearing and/or visual impairment non-correctible using prosthetic devices;
- Neurological and/or psychiatric conditions that could impede the performance of the proposed tasks (e.g., psychomotor agitation, severe behavioral symptoms);
- Participation in speech therapy within the last year.

### Cognitive assessment

All subjects were assessed through the Cambridge Examination for Mental Disorders of Older Persons with Down Syndrome and Other People with Intellectual Disabilities (CAMDEX-DS)^10,11^. This instrument comprises a structured informant interview that allows the systematic collection of information about presenting symptoms and clinical history and a direct assessment of the individual covering the following domains: Orientation, Language (comprehension and expression), Memory (learning, short-term and delayed recall), Attention, Praxis (constructional, ideomotor, and ideational), Abstract Thinking, and Perception. Emphasis is placed on establishing changes from the individual’s best previous level of functioning as informed by the principal caregiver, which is considered the most reliable index that allows the differentiation between impending cognitive decline and pre-existing impairment. Data on literacy was also obtained, and individuals were classified according to the severity of ID as mild, moderate, and severe impairment following the functional criteria of the Diagnostic and Statistical Manual of Mental Disorders, Fifth Edition (DSM-5)^12^. Cognitive diagnoses were established by a multidisciplinary team involving psychiatrists, neurologists, geriatricians, speech therapists, neuropsychologists, physical therapists, and occupational therapists, based on objective evidence of stable cognitive and behavioral status within the past five years. None of the participants underwent speech therapy after reaching adulthood.

### Language assessment

An Object and Action Naming Battery (OANB)^13,14^, a visual confrontation naming task composed of black-and-white prototypical drawings of 162 objects (nouns) and one hundred actions (verbs).

### Statistical analysis

Descriptive statistics were used to summarize and characterize the data, including measures of central tendency (mean and median) and measures of dispersion (standard deviation and interquartile range). The Shapiro-Wilk test was used to assess the data’s normality. For intergroup comparisons, participants were divided based on their level of ID and literacy (with or without functional literacy). The classification based on ID level consisted of three groups: mild, moderate, and severe; however, only one individual was labeled as having severe ID and was therefore excluded from the analyses. The classification based on literacy yielded two groups: literate and non-literate.

Fisher’s χ^2^ test was used to identify proportion differences between the groups. Student’s t-test was used to compare the distributions of a continuous variable between two independent groups (ID and literacy). Pearson’s correlation coefficient was used to assess the correlation between two continuous variables (object naming and action naming *versus* age). An alpha significance level of 0.05 was considered for all the analyses, which used Jamovi software version 2.5, 2024, and Microsoft Excel 365, 2023.

Table 1 shows the demographic characteristics of the subjects. There were no significant differences among the groups in the studied variables. The participants’ mean age was 39.5±10.2 years; range: 22–59 years.

**Table 1:** Demographic characterization of the sample.

Variable		N	%	p-value
Sex	F	14	54	0.104
	M	12	46	
Age (yrs)	50 to 59	12	46	0.610
	40 to 49	9	35	
	25 to 39	5	19	
ID level	Mild	15	58	0.328
	Moderate	10	38	
	Severe	1	4	
Education	Non-literate	16	62	0.088
	Literate	10	38	

Abbreviations: ID, intellectual disability.

Note: *Fisher’s ꭓ^2^ test.

## RESULTS

**Figure 1:**
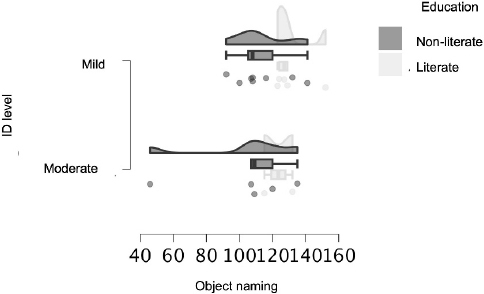
Raincloud plot showing the performance of individuals in the object naming task according to intellectual disability and educational level.

Table 2 and Figure 1 show the subjects’ performance in the object and action naming tasks according to ID level, where no significant differences were found between the groups.

**Table 2:** Performance of subjects in naming tasks according to intellectual disability level.

Variable	ID level	Mean	SD	Q1	Median	Q3	p-value
Object	Mild	117.5	21.6	107.5	123.0	130.5	0.186
	Moderate	102.8	32.5	93.5	112.0	126.0	
	Severe*	125.0	NA	125.0	125.0	125.0	
Action	Mild	50.9	20.5	45.0	52.0	62.0	0.613
	Moderate	45.9	25.3	33.0	52.0	62.7	
	Severe*	0.0	NA	0.0	0.0	0.0	

Abbreviations: NA, Not applicable; ID, intellectual disability.

Note: *one subject, excluded from statistical analysis.

**Figure 2:**
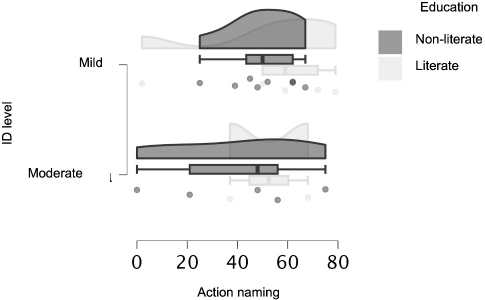
Raincloud plot showing the performance of individuals in the action naming task according to intellectual disability and educational level.

Table 3 and Figure 2 show the subjects’ performance in the object and action naming tasks according to educational level. There were no significant differences in scores between the groups.

**Table 3:** Performance of subjects in naming tasks according to educational level.

Variable	Education	Mean	SD	Q1	Median	Q3	p-value*
Object	Literate	125.4	17.2	123.0	127.0	132.0	0.041
	Non-literate	102.2	28.9	96.0	108.0	118.0	
Action	Literate	52.4	26.3	43.5	59.0	70.0	0.621
	Non-literate	47.2	20.3	40.5	50.0	61.7	

We did not find any correlation between object or action naming and age.

## DISCUSSION

To the best of our knowledge, this is the first study to address action and naming skills in a cohort of adults and aged people with DS. Our search in the United States National Library of Medicine (PubMed) database using the keywords “Down syndrome”, “naming abilities”, “naming skills”, and “naming deficits” returned only two studies involving adults with DS^15,16^, consisting in one cross sectional study and one single-case study. As such, comparison of our data with previous results in the literature was hampered.

We did not find significant differences in the naming tasks between the mild and moderate ID level groups, although individuals with moderate ID had lower scores in both object and action naming. The single cohort study found in the literature reported that individuals with DS performed better in picture naming the milder their degree of preexisting functional disability (mild>moderate>severe), irrespective of their current cognitive status (controls, mild cognitive impairment—MCI or AD); however, this study employed a modified version of the Boston Naming Test (BNT), which assessed only object naming^15^. Although no significant differences were identified in noun and verb scores among the groups based on the degree of ID, this does not imply that the degree of ID is irrelevant to linguistic performance. Previous studies suggest that individuals with milder degrees of ID may show greater potential to respond to linguistic interventions^17^. However, this relationship appears to be modulated by factors such as access to educational resources and lifelong stimulation. The lack of statistical significance in this study may reflect sample limitations or the impact of uncontrolled variables, such as differences in social support, rather than a true independence between the variables.

On the other hand, literacy proved to be a factor influencing linguistic performance only for object naming, but not for verbs. Literate participants scored higher in object naming, pointing the positive role of formal education in linguistic development^18^. However, this effect was not observed for verb naming. This contrast between nouns and verbs underscores the complexity of the linguistic processes involved and suggests that schooling may unequally influence specific areas of language. As noted by some authors^19,20^, the cognitive processes associated with verb retrieval may rely more on semantic and syntactic skills than on the direct impact of literacy.

Correlation analyses showed that age was not associated with performance in any of the naming tasks. The absence of correlation between age and linguistic scores in our study suggests that chronological aging alone may not be a determinant factor in linguistic decline among adults with DS. However, it is important to consider that chronological age may mask the effect of other factors, such as the progression of comorbidities or the accumulation of barriers and learning difficulties, which often accompany aging in individuals with DS. Bayen et al.^21^ emphasize that dementia is a predominant factor contributing to cognitive and linguistic decline in adults with DS. To substantiate that, we reference the study by Kledaras et al.^16^, which documented the progressive impairment in naming skills of a 59-year-old male with DS who developed dementia over a period of 20 months follow-up.

The interaction between the degree of ID and contextual factors, such as schooling and social support, appears to play a relevant role in the linguistic performance of individuals with DS. This interaction highlights the contrast between intrinsic characteristics, such as the degree of ID, and extrinsic factors, such as the educational environment, in determining linguistic performance. In this context, the historical-cultural perspective emphasizes that cognitive and linguistic development results from the dynamic interaction between the individual and their social environment, suggesting that intrinsic and contextual factors are intricately interconnected^22^. Chapman and Hesketh^23^ further argue that, while cognitive deficits may limit linguistic development, the social and educational environment can either mitigate or amplify these effects, depending on the level of stimulation provided.

The findings of this study reaffirm the importance of understanding the relationship between contextual and individual factors in linguistic performance, particularly in populations with intellectual disabilities and associated aging. While factors such as literacy directly influenced linguistic performance, others such as age and degree of ID seem to depend on more complex interactions with external variables to exert their impact^22^. Additional studies are needed to explore the impact of sociocultural factors, educational stimuli, and health conditions in different subgroups, which will allow for a deeper understanding of the dynamics involved. Furthermore, the use of specific tools, such as the OANB, enables a more detailed and differentiated analysis of linguistic abilities, contributing to the identification of potential interventions to optimize the performance of this population.

## Data Availability

The datasets generated and/or analyzed during the current study are available from the corresponding author upon reasonable request.
